# Lasting personality pathology following exposure to severe trauma in adulthood: retrospective cohort study

**DOI:** 10.1186/s12888-018-1975-5

**Published:** 2019-01-03

**Authors:** Jasna Munjiza, Dolores Britvic, Mike J. Crawford

**Affiliations:** 1 Centre for Psychiatry, Faculty of Medicine, Imperial College London, Hammersmith Campus, 7th Floor Commonwealth Building, Du Cane Road, London, W12 0NN UK; 2 Central and North West London NHS Foundation Trust, London, UK; 3 Department of Psychiatry, School of Medicine Split, University of Split, Split, Croatia

**Keywords:** Late-onset personality pathology, Severe trauma, PTSD, Personality disorder, War

## Abstract

**Background:**

Early exposure to trauma is a known risk factor for personality disorder (PD), but evidence for late-onset personality pathology following trauma in adults is much less clear. We set out to investigate whether exposure to war trauma can lead to lasting personality pathology in adults and to compare the mental health and social functioning of people with late–onset personality problems with those with PD.

**Methods:**

We recruited patients who scored positively on the International Personality Disorder Examination (IPDE) in southern Croatia 15 years after the Croatian war of independence and used a semi-structured interview to establish when the person’s personality-related problems arose. All participants also completed Harvard Trauma Questionnaire, and measures of mental health and social functioning.

**Results:**

Among 182 participants with probable personality disorder, 65 (35.7%) reported that these problems started after exposure to war-trauma as adults. The most prevalent personality problems among those with late-onset pathology were borderline, avoidant, schizotypal, schizoid and paranoid. Participants with late-onset personality pathology were more likely to have schizotypal (75.4% vs. 47.3%) and schizoid traits (73.8% vs. 41.1%) compared to those with PD. Participants with late-onset personality pathology were three times more likely to have complex personality pathology across all three DSM-IV clusters compared to those with PD (OR = 2.96, 95% CI 1.54 to 5.67) after adjusted for gender and marital status. The prevalence of depression and social dysfunction were as high among those with late-onset personality pathology as among those with personality disorder.

**Conclusion:**

Retrospective accounts of people with significant personality pathology indicate that some develop these problems following exposure to severe trauma in adulthood. Personality-related problems which start in adulthood may be as severe as those that have an earlier onset. These findings highlight the long term impact of war trauma on the mental health and have implications for the way that personality pathology is classified and treated.

## Background

The effects of exposure to trauma in childhood have repeatedly been linked to the development of maladaptive personality traits and personality disorders [1–4]. In contrast, much less is known about personality related problems that may arise in adulthood. Following military conflicts in South East Asia in the 1960s and 1970s, clinical and research interest in the psychological sequelae of exposure to severe trauma expanded enormously. This led to introduction of a new diagnostic category of Post Traumatic Stress Disorder (PTSD) first in DSM-III [5] and later in the ICD-10 [6]. For the following 40 years, most research related to trauma in adults has focused on different aspects of PTSD including presenting symptomatology, prognosis and response to treatment indicating that both mental and physical illness comorbidity was high in the PTSD patients [7–11]. However, the experts in the field of trauma also argued that the existing PTSD diagnostic criteria failed to capture some of the enduring psychological problems experienced by adults exposed to major trauma [12, 13]. As a result, a new diagnostic category named Enduring Personality Change after Catastrophic Experience (EPCACE, F62.0) was introduced to ICD-10 [6]. This condition is defined as a change of at least 4 years duration in how one perceives, relates to or thinks about the environment and self following the exposure to catastrophic trauma. It excludes individuals with a pre-trauma history of personality disorder and therefore is meant to be used solely in cases with the late-onset personality pathology. Despite several attempts to include EPCACE and ‘complex PTSD’ in both DSM IV and DSM-5, this proposal was turned down [14, 15].

A recent systematic review of extant literature [16] suggested that a proportion of healthy adults who are exposed to severe trauma appear to go on to develop significant personality problems. However, the review also identified the absence of high quality studies with appropriate controls and the lack of studies that investigated pre-morbid personality pathology. Without assessing the latter, the fundamental distinction between personality disorder (i.e. pre-trauma personality problems) and personality pathology that develops during exposure to severe trauma in adulthood cannot be made.

Inconsistencies between ICD and DSM classifications reflect genuine uncertainties about whether long standing personality problems can result from exposure to severe trauma in adults. We set out to investigate whether exposure to severe war-related trauma is associated with long-term personality pathology in adults. Therefore our primary aim was to identify among adults with significant personality pathology, the proportion of people who had late-onset personality problems. The secondary aim was to compare the mental health and social functioning of people with personality disorder with those of late-onset personality pathology.

## Methods

The methods including the recruitment process have been described in detail elsewhere [17]. Briefly, in this case-control study [17] cases met the threshold for significant personality-related problems using the International Personality Disorder Examination (IPDE) [18] whilst controls did not. Out of 268 patients who participated in the original case-control study [17], 182 patients met the threshold for having significant personality pathology. High levels of exposure to severe war trauma among the local population in the region of Southern Croatia 15 years prior to data collection, made this an appropriate setting in which to examine whether these experiences are associated with late-onset personality pathology or they are a consequence of pre-trauma personality disorder.

### Participants

Participants were recruited from outpatient and inpatient mental health services at the University Hospital of Split (southern Croatia). We asked clinicians to refer people who were primarily being treated for personality-related problems and had lived in Croatia during the 1991–95 war. Only participants who signed informed consent were included in the study. People suffering from an acute psychotic episode, chronic psychotic illness or from personality change due to organic brain damage were excluded. Data was collected over a period of 12 months and the recruitment was completed by October 2011. The study was approved by the University Hospital Split Ethics Committee and the School of Medicine Ethics Committee, University of Split (Ref No: 2181–198–03-04/10/10/0017) prior to the start of data collection.

### Measures

All participants were asked to complete a set of self-report questionnaires followed by a semi-structured clinical interview.

The primary outcome was the assessment of personality-related pathology assessed with the International Personality Disorder Examination (IPDE) measure [18]. The self-report screen contains 77 items measuring personality pathology according to the DSM-IV and includes 10 PD subcategories. The items related to each PD subgroup are interspersed between the 10 personality categories to reduce the likelihood of participants choosing desirable answers. A more rigorous approach for scoring participants answers was used in this study which meant that a score of three and below meant ‘negative’ for a PD subcategory whilst a score of four and above meant ‘positive’ for that personality subgroup. The IPDE has good inter-rater reliability (071–0.91) and intertemporal reliability (0.55–0.84) [18].

We used several secondary outcome measures. The Harvard Trauma Questionnaire (HTQ) was used to assess war-related trauma and symptoms of post-traumatic stress [19]. This widely translated self-report measure has been also culturally adapted and extensively used, tested and validated in traumatised civilian populations and war veterans throughout the world including the communities of the former Yugoslavia [20, 21]. The measure includes 16 items derived from DSM IV PTSD criteria based on the three sub-domains: re-experiencing traumatic events, avoidance and increased arousal. A cut off score of ≥2.5 is considered to be “checklist positive” for PTSD. Although some previous research in the former Yugoslavia recommended a cut-off score of ≥2.0 for PTSD ‘positive’ cases [21], we used a more conservative cut off point of ≥2.5 to make our findings comparable to wider international communities.

We used the Hopkins Symptom Checklist-25 (HSCL-25) to assess symptoms related to depression and anxiety [20]. This 25-item self-report measure is divided in two parts: a 10-point anxiety scale and a 15-item scale of depressive symptoms which are consistent with the DSM diagnoses of generalized anxiety disorder and major depression. The HSCL-25 has been translated and culturally adapted for the communities of former Yugoslavia [21]. The recommended cut-off point of ≥1.75 was used for the HSCL-25 diagnoses of depression and anxiety [20].

The Social Functioning Questionnaire (SFQ) is an eight-item self-report measure that was used to assess participants’ levels of social functioning. Each of the eight items is rated on a scale from 0 to 3, with higher scores indicating more dysfunction. This robust measure has been found to have good inter-rater reliability and construct validity [22]. Scores of 10 and above on the SFQ screen indicate poor social functioning and are positively associated with a diagnosis of personality disorder.

To assess the onset of personality difficulties reported on the IPDE we used a face to face semi-structured clinical interview. We used this interview to clarify when personality-related problems started, whether they were present during participants’ late childhood and/or adolescence or if these difficulties started later in their adult life. Before the interview, the researcher briefly checked each participant’s responses on the IPDE scale, and then asked them when they first became aware of the personality difficulties that they endorsed on the IPDE measure. Finally participants were asked a series of questions about their childhood and adolescence. These included information on problems in school (being bullied, receiving cautions, being expelled) and at home (running away, being in trouble with the police, relationship with parents). Participants were also asked about their history of close relationships, employment and abuse of substances before the war.

As we were interested in studying the exposure to severe trauma and its impact on potential personality change in adults, and in the absence of an established definition of this concept, we have decided to define severe (catastrophic) trauma based on the ICD-10 description of catastrophic stress (WHO, 1992) and the findings from a survey of trauma experts [23]. This process of defining the catastrophic trauma has been described in detail elsewhere [17]. Briefly, it was assumed that severe (catastrophic) trauma would involve prolonged exposure to life-threatening circumstances with imminent possibility of being killed (for example exposure to war trauma, concentration camp experience, being tortured, hostage situations and sexual assault). Two authors (JM and MC) independently assessed 47-items of HTQ trauma events (Part I) and selected those items in the HTQ that they thought would meet the criteria for severe trauma resolving any disagreements by further discussions. Out of the 47 war-related traumatic events listed in the Harvard Trauma Questionnaire, 17 items (36%) were considered to be of the severity that could be described as ‘severe war-related trauma’ [17].

**Fig. 1:**
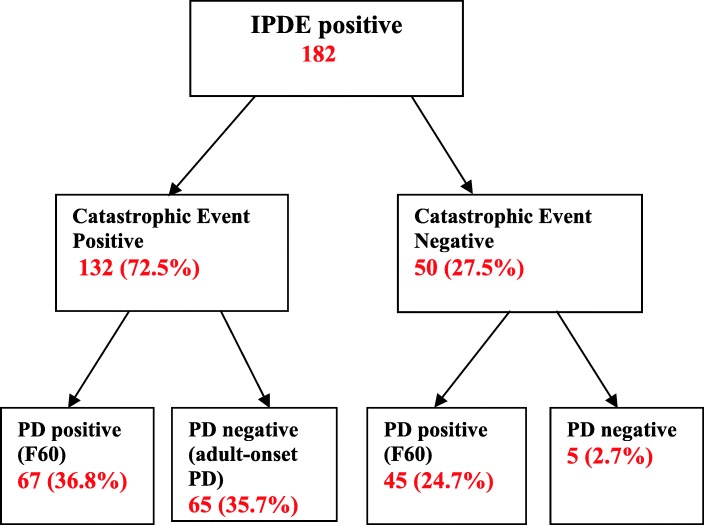
Flowchart of participants’ groups based on IPDE scoring, exposure to catastrophic trauma and presence of personality disorder. IPDE positive (based on scores from the International Personality Disorder Examination Questionnaire) - participants scoring positive on IPDE; IPDE negative - participants scoring negative on IPDE. Catastrophic event (positive or negative) based on the 17 items from the Harvard Trauma Questionnaire. PD positive – pre-trauma personality pathology present; PD negative – pre-trauma personality pathology absent – based on the semi-structured interview

By combining the findings from the three variables: IPDE status (positive vs. negative); exposure to catastrophic trauma (positive vs. negative) and the outcome of the clinical interview (pre-existing personality pathology present vs. pre-existing personality pathology absent) each participant was categorized as having pre-existing personality pathology (suggestive of personality disorder), personality pathology with onset in adulthood or no significant personality pathology (Fig. 1). In other words, a group of patients that were IPDE positive and exposed to catastrophic trauma, but had no evidence of pre-trauma personality pathology, were considered to have developed personality problems following exposure to catastrophic war-related trauma in adulthood. All clinical interviews were conducted by one researcher (JM).

### Data analysis

The first analyses compared the demographics of PD and late-onset PD patients. The chi-square test was used to for the categorical variables, whilst the unpaired t-test was used for continuous variables following a Normal distribution.

Subsequently the two groups were compared in terms of measures of personality pathology, the number of PD criteria clusters met and measures of mental health and social function. For each analysis, an unadjusted comparison of the outcomes between groups was first performed. Subsequently, the differences were re-examined, adjusting for demographic factors found to differ between groups from the initial analyses. Binary outcomes (measures of personality pathology and mental health/social function) were analysed using binary logistic regression. The number of PD criteria clusters met was an ordinal measure, and was analysed using ordinal logistic regression.

## Results

Out of 268 patients recruited into the case-control study [17], 182 patients met the threshold for significant personality pathology using the International Personality Disorder Examination (IPDE) questionnaire. Among them, 132 (72.5%) reported experiencing one or more severe war-related (catastrophic) event [17].

Among the 182 the IPDE positive patients, 112 (61.5%) had evidence of PD prior to exposure to war trauma and 70 (38.4%) did not. Out of 70 patients, five (2.74%) did not report exposure to any catastrophic event and had no personality related pathology indicating PD before the 1991–95 war. These five participants were excluded from further analysis. The remaining 65 participants who reported no personality related problems during late childhood and/or adolescence which suggested development of personality pathology, but scored positive for catastrophic trauma and were IPDE positive, were considered to develop late-onset personality pathology.

Demographic characteristics of PD patients and patients with late-onset personality pathology are shown in Table 1.

**Table 1:** A comparison of demographic factors in PD patients and late-onset personality pathology patients

Variable	PD patients *N* = 112	Late-onset PD *N* = 65	Mean or proportion difference (95% CI of the difference)	*P*-value
Age - Mean (SD)	44.18 (10.16)	46.51 (8.37)	2.33 (−0.61 to 5.27)	0.12
Gender N (%)				
Male	63 (56.3.)	55 (84.6.)	0.28 (0.15 to 0.40)	**< 0.001**
Female	49 (43.8)	10 (15.4)		
Ethnicity N (%)^a^				
Croatian	108 (96.4)	63 (96.9)	0.01 (− 0.07 to 0.06)	1.00
Other	4 (3.6)	2 (3.1)		
Education (%)				
No qualifications	11 (9.8)	4 (6.2)	0.04 (−0.06 to 0.12)	0.43
A levels/vocational	78 (69.6)	51 (78.5)	0.09 (−0.05 to 0.21)	
University	23 (20.5)	10 (15.4)	0.05 (−0.07 to 0.16)	
Marital status N (%)				
Single	27 (24.1)	10 (15.4)	0.09(−0.04 to 0.20)	**0.002**
Married/with partner	65 (58.0)	53 (81.5)	0.24 (0.09 to 0.35)	
Divorced/separated/widow	20 (17.9)	2 (3.1)	0.15 (0.05 to 0.23)	
Recruitment area N (%)^a^				
Outpatient	106 (94.6)	64 (98.5)	0.04 (− 0.03 to 0.10)	0.43
Inpatients	6 (5.4)	1 (1.5)		

^a^ Analysis using Fisher’s exact test

There was no significant difference between the two groups in terms of their age, ethnicity, educational attainment or inpatient/outpatient status, but participants with late-onset personality disorder were more likely to be male (85% vs. 56%, X^2^ (1, *n* = 177) = 13.39, *p* < 0.001) and married or living with a partner (82% vs. 58%, X^2^ (2, *n* = 177) =11.96, *p* = 0.002).

### Characteristics of personality-related problems in late-onset personality pathology patients (according to DSM-IV criteria)

The proportion of people with PD and late-onset personality problems meeting the threshold for different personality sub-categories are presented in Table 2. The findings suggest that schizoid and schizotypal traits were more common in the late-onset PD group than the PD subgroup, whereas histrionic trait was significantly less common in this group. The differences between groups for these variables remained after adjusted for demographics found to vary between groups. There were no differences in the two groups for the other measures.

**Table 2:** A comparison of personality pathology between PD and late-onset personality disorder patients

Variables	PD	Late-onset PD	Unadjusted		Adjusted^a^	
	*N* (%)	*N* (%)	OR (95% CI)^b^	*P*-value	OR (95% CI)^b^	*P*-value
Paranoid	61 (54%)	44 (68%)	1.75 (0.92 to 3.32)	0.09	1.62 (0.82 to 3.21)	0.16
Schizoid	46 (41%)	48 (74%)	4.05 (2.07 to 7.91)	< 0.001	3.65 (1.79 to 7.43)	< 0.001
Schizotypal	53 (47%)	49 (75%)	3.41 (1.74 to 6.70)	< 0.001	2.78 (1.35 to 5.71)	0.005
Histrionic	41 (37%)	9 (14%)	0.28 (0.12 to 0.62)	0.002	0.30 (0.13 to 0.70)	0.005
Antisocial	16 (14%)	13 (20%)	1.50 (0.67 to 3.36)	0.32	1.02 (0.43 to 2.41)	0.96
Narcisistic	35 (31%)	17 (26%)	0.78 (0.39 to 1.54)	0.47	0.77 (0.37 to 1.59)	0.48
Borderline	85 (76%)	56 (86%)	1.98 (0.86 to 4.52)	0.11	1.55 (0.64 to 3.73)	0.33
Compulsive	69 (62%)	39 (60%)	0.93 (0.50 to 1.75)	0.83	0.95 (0.49 to 1.86)	0.88
Dependent	49 (44%)	19 (29%)	0.53 (0.28 to 1.02)	0.06	0.58 (0.29 to 1.16)	0.12
Avoidant	83 (74%)	56 (86%)	2.17 (0.96 to 4.94)	0.06	1.73 (0.72 to 4.18)	0.22

^a^Adjusted for gender and marital status

^b^Odds ratios presented as odds of outcome in Late-onset PD subgroup relative to odds in PD subgroup

We further examined the relationship between the two groups (PD and late-onset personality problems) according to the number of cases meeting diagnostic criteria of personality traits across the DSM-IV conceptual clusters. Table 3 displays the proportions of participants meeting diagnostic criteria for one, two and three clusters, including the crude and adjusted odds ratios with 95% confidence intervals. The findings suggest a significant difference in numbers of PD cluster criteria met between groups. Participants in the late-onset personality pathology group were more likely to meet the criteria across all three DSM-IV clusters. Additionally, we compared the prevalence of individual IPDE scores reported by PD group and late-onset personality disorder patients. More than 80% of the patients with late-onset disorder reported having persistent feelings of emptiness, frequent mood changes and having anger regulation problems. Equally high proportion of them reported avoidance of social interactions and preferring doing things by themselves to minimise contacts with others. More than two-thirds reported feeling ‘cold and detached’ and having difficulties showing emotions. The same proportion of them did not feel they could trust others and more than 80% felt that they have been treated unfairly by others including experiencing attacks on their character and reputation. Impulsiveness and identity problems were reported by more than 60% of participants in this group. More than half of them reported feeling ‘odd and eccentric’, being rigid and inflexible and sensitive to criticism.

**Table 3:** Comparison between PD and late-onset disorder groups based on number of participants meeting diagnostic criteria across one or more DSM-IV clusters

Number of DSM-IV Clusters	PD group	Late-onset PD	Unadjusted		Adjusted ^+^	
	N (%)	N (%)	OR (95% CI) *	*P* value	OR (95% CI) *	*P* value
Meeting criteria of 1 PD cluster	28 (25%)	8 (12%)	3.54 (1.91 to 6.56)	< 0.001	2.96 (1.54 to 5.67)	**0.001**
Meeting criteria of 2 PD clusters	63 (56%)	25 (38%)				
Meeting criteria of all 3 PD clusters	21 (19%)	32 (49%)				

+ Adjusted for gender and marital status

* Odds ratios presented as odds of being in the next highest outcome category for Late-onset PD subgroup relative to odds in PD subgroup

### Mental health and social functioning

A summary of the results on mental health, experience of suicidal thoughts, social functioning and employment in both groups is given in Table 4. The size of difference between groups are reported as the odds of the outcome in the late-onset personality pathology group relative to the odds in PD group.

**Table 4:** Comparisons of mental health and social function between late-onset personality pathology patients and PD patients

Variables	Late-onset PD N (%)	PD N (%)	OR (95% CI) ^a^	*P*-value	Adjusted OR (95% CI) ^a^	*P*-value
Depressive symptoms^1^	62 (95%)	101 (90%)	2.25 (0.60 to 8.39)	0.23	1.70 (0.42 to 6.86)	0.45
Anxiety symptoms^1^	60 (92%)	89 (79%)	3.10 (1.12 to 8.61)	0.03	2.42 (0.83 to 7.04)	0.11
PTSD symptoms^2^	51 (82%)	51 (51%)	4.45 (2.08 to 9.53)	< 0.001	2.94 (1.30 to 6.67)	**0.01**
Social dysfunction^3^	56 (88%)	78 (74%)	2.51 (1.07 to 5.92)	0.04	2.28 (0.90 to 5.74)	0.08
Employed^1^	11 (17%)	29 (26%)	0.58 (0.27 to 1.26)	0.17	0.82 (0.35 to 1.88)	0.63
Suicidal thoughts^1^	44 (68%)	53 (47%)	2.33 (1.23 to 4.42)	0.009	1.96 (0.99 to 3.86)	**0.05**

^a^Odds ratios presented as odds of outcome in Late-onset PD subgroup relative to odds in PD subgroup

(Denominators in each of these categories varied according to the completeness of the related scales/records; ^1^Late-onset PD = 65, PD = 112; ^2^ Late-onset PD = 62, PD = 100; ^3^ Late-onset PD = 64, PD = 106)

The results suggested that anxiety, PTSD, social functioning and suicidal thoughts significantly differed between groups when the demographics of the patients were not considered in the analysis (unadjusted analysis). For all variables where there was a difference, the outcomes were more likely in the late-onset personality pathology group than in the PD group. After adjusting for gender and marital status, significant differences in PTSD and prevalence of the suicidal thoughts between the two groups remained.

## Discussion

The main aim of this study was to investigate whether exposure to war trauma could lead to personality pathology in adults. Having found that people who had personality-related problems were more likely to have been exposed to war trauma [17], we then set about examining the role that the war trauma may have played in the onset of their condition. To do this, we examined whether people had evidence of personality-related problems prior to their exposure to war trauma and compared the characteristics of the people with PD with those whose personality-related problems appeared to follow exposure to trauma in adulthood. Among 182 cases who were IPDE screen positive, 65 participants (35.7%) had no history of pre-trauma personality pathology, suggesting development of personality problems in adulthood which followed their exposure to severe trauma.

Aspects of disordered personality were assessed in this study using the IPDE 77-item questionnaire. The threshold for avoidant and borderline traits was reached by 86.2% of patients with late-onset personality pathology, schizotypal by 75.4%; schizoid by 73.8%, paranoid by 67.7%, and anankastic by 60%, but only schizoid (73.8% vs 41.1%) and schizotypal traits (75.4% vs. 47.3%) were significantly more frequent in patients with late-onset personality problems than in PD patients.

An additional and important finding was that people with late-onset personality psychopathology following exposure to severe war trauma were three times more likely to meet the criteria for personality problems across all three DSM-IV conceptual clusters than the PD group. These findings suggest that the complexity and degree of personality related problems in patients with late-onset personality pathology is greater than in those with PD alone. The finding indicating that a considerable proportion of patients met threshold for two or more personality traits is consistent with prior research which suggested that most people with a diagnosis of personality disorder ‘do not fit’ into a single personality disorder subcategory. Instead, they tend to meet criteria for two or more subcategories within one cluster or across two or even all three clusters [24, 25].

When compared to PD patients, the late-onset personality pathology group had equally poor mental health and social functioning and similarly high rates of unemployment. Late-onset personality pathology patients were three times more likely to suffer from PTSD than their PD counterparts. These results indicate a strong need for trauma-focused therapies to reduce PTSD-related symptomatology, although these may not be readily available in countries devastated by war. Furthermore, they reported significantly more suicidal thoughts than the PD group (68% vs 47%). These results provide evidence that people with late-onset personality pathology have levels of emotional distress that are as high, if not higher, than patients with personality disorder. These symptoms appear to be enduring and impact on interpersonal functioning for more than 15 years following exposure to catastrophic trauma.

### Strengths and limitations

The study has several strengths. It assessed for pre-trauma personality pathology, which allowed to separate those who developed personality related problems following the traumatic war experience in adulthood from patients who had pre-existing personality disorder. In other words, this study addressed a confound encountered in much of the previously published literature [16] as very few studies assessing the impact of catastrophic trauma, investigated presence of pre-morbid personality pathology. Although in our study this information was gathered retrospectively and thus prone to a recall bias, this issue was particularly explored during the semi-structured clinical interview whose sole purpose was to establish the onset of personality-related pathology. We conducted the study in a war-affected area which helped to recruit a large number of participants who had been exposed to war trauma.

However, our study also has a number of limitations. Recall bias if one of them, which potentially could have influenced the participants’ accounts of traumatic war experience. We assessed war trauma (the exposure of interest) equally in both groups by using a standardised self-report measure Harvard Trauma Questionnaire with no additional prompting from researchers. The use of a self-report measure to assess personality pathology is an additional limitation. Validity of personality screening questionnaires has been questioned when compared to semi-structured interviews [26]. However, most of the cases recruited in this study already had a clinical diagnosis of personality disorder (F60.0 – F60.9) or personality change (F62.0). A further important limitation that needs to be considered is the lack of collateral history to validate participants’ accounts of pre-trauma functioning and personality pathology. Although participants had opportunity to describe any other past trauma by which they felt affected whilst completing the HTQ questionnaire, we did not explore this during the interviewing process as its main focus was to establish pre-war personality pathology. However, most of the participants who answered this HTQ question chose to describe war-related incidents.

### Implications for diagnostic classifications and further research

The findings from this study have two important implications for the classification of mental disorders. Firstly, they suggest that late-onset personality disorder is a valid diagnostic category and the development of personality pathology should not be restricted to those who experienced personality problems during childhood and adolescence as the current two major disease classification systems suggest [6, 27]. In other words, where there is incidence of severe trauma, a person’s personality can change at a later point in life. Secondly, findings from this study suggest that the personality psychopathology of people experiencing catastrophic trauma in adulthood is a complex phenomenon. The elicited personality characteristics are multifarious and include personality features from all three DSM-5 conceptual clusters. These findings could help to increase our understanding of why these patients have a very complex clinical presentation and a limited response to treatment which has been observed in clinical practice [28–31]. Furthermore, consistent with the findings from a recent review [16], the results from this study suggest that the current ICD-10 diagnostic criteria for the diagnosis of enduring personality change after a catastrophic experience (F62.0) should be revised to include a much wider range of psychopathology such as affect regulation difficulties, anger modulation problems, impulsivity, feelings of being detached, being oversensitive to criticism, rigidity and inflexibility in interpersonal interactions, increased suicidality, and self-identity problems as none of these are currently included in the ICD-10 diagnostic classification.

The proposal to include either ‘complex PTSD’, DESNOS or enduring personality change after catastrophic trauma as a separate diagnostic entity were rejected by DSM IV and DSM 5 due to insufficient research evidence to support these concepts as a separate diagnosis in the DSM [14, 15].

The ICD-11 Working Group reviewing PTSD classification proposes the introduction of a new diagnostic term named ‘Complex post-traumatic stress disorder’ which they recommend should be used instead of EPCACE to diagnose psychopathology arising from ‘severe and prolonged stressors usually involving several or repeated adverse events’ [32]. However, the proposed diagnostic criteria clearly overlap with the symptomatology experienced by people with personality disorder. The lack of clarity between the two conditions (PD and complex PTSD) could potentially lead to further inconsistencies in both research and clinical practice.

Perhaps similarly to the current proposals for reclassifications of personality disorder in ICD11, which focus more on the severity of personality problems [33–35], personality pathology following trauma in adulthood could be looked at dimensionally based on the severity of the observed pathology. The ICD-11 working group also propose introducing a ‘late onset’ specifier for PD cases where personality disturbance originates in adulthood and there is no evidence of personality related problems before age of 25 years [35].

## Conclusion

Our findings suggest that a proportion of people who were exposed to severe war-related trauma developed personality-related pathology in adulthood. Patients with late-onset personality problems had equally poor mental health and social functioning when compared to PD patients. These findings highlight the long term impact of war trauma on the mental health and have implications for the way that personality pathology is classified and treated. More research needs to be done to develop appropriate assessment and treatment for people who have significant personality-related problems that develop after childhood and adolescence. Additionally, further research is needed to increase our understanding of any potential risk factors that may contribute to the development of late-onset personality pathology.

## Abbreviations

HSCL-25: Hopkins Symptom Checklist-25
HTQ: Harvard trauma questionnaire
IPDE: International Personality Disorder Examination
PTSD: post-traumatic stress disorder
SFQ: Social Functioning Questionnaire
